# Amino acids at position 5 in the peptide/MHC binding region of a public virus‐specific TCR are completely inter‐changeable without loss of function

**DOI:** 10.1002/eji.202249975

**Published:** 2022-10-19

**Authors:** Wesley Huisman, Melanie de Gier, Lois Hageman, Alina S. Shomuradova, Didier A.T. Leboux, Derk Amsen, J.H. Frederik Falkenburg, Inge Jedema

**Affiliations:** ^1^ Department of Hematology Leiden University Medical Center The Netherlands; ^2^ Department of Hematopoiesis Sanquin Research and Landsteiner Laboratory for Blood Cell Research Amsterdam The Netherlands; ^3^ Laboratory for Transplantation Immunology National Research Center for Hematology Moscow Russia

**Keywords:** amino acids, T‐cell receptors, public TCRs, VDJ‐recombination, virus‐specific T cells

## Abstract

Anti‐viral T‐cell responses are usually directed against a limited set of antigens, but often contain many T cells expressing different T‐cell receptors (TCRs). Identical TCRs found within virus‐specific T‐cell populations in different individuals are known as public TCRs, but also TCRs highly‐similar to these public TCRs, with only minor variations in amino acids on specific positions in the Complementary Determining Regions (CDRs), are frequently found. However, the degree of freedom at these positions was not clear. In this study, we used the HLA‐A*02:01‐restricted EBV‐LMP2^FLY^‐specific public TCR as model and modified the highly‐variable position 5 of the CDR3β sequence with all 20 amino acids. Our results demonstrate that amino acids at this particular position in the CDR3β region of this TCR are completely inter‐changeable, without loss of TCR function. We show that the inability to find certain variants in individuals is explained by their lower recombination probability rather than by steric hindrance.

## Graphical abstract

In this study, the HLA‐A*02:01‐restricted EBV‐LMP2^FLY^‐specific public TCR was used as model and the highly‐variable position 5 of the CDR3β sequence was substituted with all 20 amino‐acids. Our results demonstrate that amino‐acids at this particular position in the CDR3β region of this TCR are completely inter‐changeable, without loss of TCR function

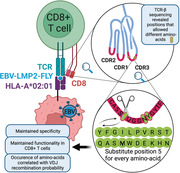

## Introduction

Human virus‐specific CD8^pos^ T cells express a heterodimeric alpha(α)/beta(β) T‐Cell Receptor (TCR) that specifically recognizes a viral‐peptide in the context of a human leukocyte antigen (HLA)‐class‐I molecule. The TCR‐β chains have highly variable sequences due to recombination of the Variable (V) β genes with their corresponding Diversity (D) and Joining (J) genes and the nucleotides that are added at the V‐D‐J junctions [1]. The TCRα‐chains are generated similarly, with the exception of a D gene, resulting in V‐J reading frames [2]. Gene segment rearrangements could potentially generate a repertoire of 10^15^–10^20^ unique TCRs(3). The Complementary Determining Regions (CDRs) are the sequences in the TCR that form loops and are responsible for the interaction with the peptide and HLA molecule. The CDR1 and CDR2 regions are fixed within the germline sequence of the V gene and their variability is based on the different V‐genes [4, 5]. The sequence at the recombined V‐D‐J and V‐J regions encodes for the CDR3 region, which is highly variable and greatly determines the specificity of the TCR. Although anti‐viral T‐cell responses are usually directed against a limited set of viral epitopes [6], they often contain many T cells expressing different TCRs. However, we and others have shown that identical TCRs directed against specific viral peptides can be found in different individuals, known as public TCRs [7, 8, 9, 10].

It was recently shown by us and others that anti‐viral TCR‐repertoires also comprised receptors that were highly similar to public TCRs and were restricted to the same HLA molecule and specific for the same peptide [9, 11‐15]. These highly‐similar TCRs were different from the public TCR on specific positions in the CDR3 regions. Key conserved residues in these CDR3 regions were identified as essential elements of TCR recognition [13]. High variability of amino acids was often found on positions at the site of V to D or D to J recombination, but not all 20 amino acids were found at such a promiscuous position [12, 15]. We recently identified an HLA‐A*02:01‐restricted public TCR with CDR3α [CATEGDSGYSTLTF] and CDR3β [CASSYQGGNYGYTF] that is specific for EBV‐LMP2^FLY^ and was found in 9 out of 11 EBV^pos^ HLA‐A*02:01^pos^ individuals [16]. In total, 10 other highly‐similar TCRs were found, that were also specific for EBV‐LMP2^FLY^, with amino acids being only different on position 5 of the CDR3β‐sequence [CASSxQGGNYGYTF]. As not all amino acids were found on this position, it is conceivable that specific rules may still limit the degree of sequence freedom in this location of the CDR3 region. Indeed, evidence was recently provided that positively charged and hydrophobic amino acids in CDR3β sequences are disfavoured and that TCRs with cysteines in their CDR3 peptide‐binding regions are clonally deleted [11]. To test why such TCRs are disfavoured, fully human TCRs could be sequenced, altered to express a cysteine in their CDR3‐peptide‐binding region, and transduced into primary T cells. However, transducing fully humanized TCRs into primary T cells might result in the mispairing with endogenous TCRα and/or TCRβ‐chains, resulting in unexpected specificity/reactivity [17] and competition for the TCR complex. Mutations of single amino acids to cysteines in each TCR Constant domain can lead to additional disulfide bonds, increasing preferential pairing [18]. However, when human TCR Constant regions were replaced by their murine counterparts, an even further decreased expression of the hybrid TCRs was noted [19].

In this study, we used the public TCRαβ sequence specific for HLA‐A*02:01‐restricted EBV‐LMP2^FLY^ as a model and systematically replaced the amino acid at the highly‐variable position 5 in the CDR3β sequence of this public TCR with all amino acids to investigate whether specific rules apply to this highly‐variable position. We transduced all 20 artificially generated TCRβ‐sequence variants with the public TCRα‐sequence in primary CD8^pos^ T cells of healthy EBV^neg^ HLA‐A*02:01^neg^ individuals. We found that all variants remained specific for EBV‐LMP2^FLY^ without major differences in functionality. Our results illustrate that amino acids on position 5 of this public TCR are completely interchangeable. We show that limitations in recombination probabilities likely restrict the appearance of specific amino acids in the EBV‐LMP2^FLY^‐specific TCR repertoires.

## Results

### Artificially generated TCR‐sequences keep specificity despite amino acid changes in the CDR3β‐region

**Figure 1:**
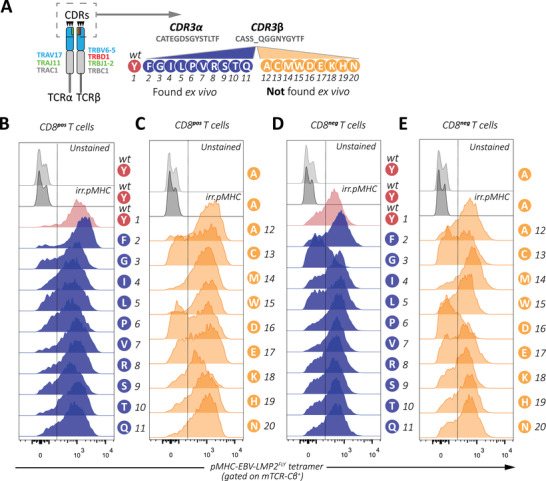
Amino acid substitutions in the complementary determining region 3 of HLA‐A*02:01‐restricted EBV‐LMP2‐specific TCRs show overall maintenance of specificity in primary CD8^pos^ and CD8^neg^ T cells. Primary CD8^pos^ and CD4^pos^ (CD8^neg^) T cells were isolated using MACS. TCR‐transduced primary T cells were purified based on expression of murine‐TCR‐Cβ using MACS and expanded. Double positive CD4 ^pos^CD8 ^pos^ T cells were not observed in the analyses. **A)** The T‐cell receptors of FACSorted memory EBV‐LMP2^FLY^‐specific T‐cell populations were sequenced from peripheral blood of 11 different donors. The identical (public) HLA‐A*02:01‐restricted EBV‐LMP2^FLY^‐specific TCR with the CDR3β sequence CASSYQGGNYGYTF was found in 9 out of 11 individuals (Red). CDR3β sequences highly‐similar to this public sequence were found that contained either one of 10 different amino acids on position 5 of the CDR3β‐sequence (Blue). Of all possible amino acids, 9 amino acids on position 5 of HLA‐A*02:01‐restricted EBV‐LMP2^FLY^‐specific CDR3β sequences, were not found *ex vivo* (orange). **B** and **C**) Shown are histograms of specific HLA‐A*02:01/pMHC‐EBV‐LMP2^FLY^ tetramer (red, blue and orange) or irrelevant HLA‐A*02:01/pMHC‐CMV‐pp65^NLV^ tetramer (black) stainings of CD8^pos^ T cells transduced with TCRs that were found (**B**) or that were not found *ex vivo* (**C**). **D** and **E**) Shown are histograms of specific HLA‐A*02:01/pMHC‐EBV‐LMP2^FLY^ tetramer (Red, Blue and Orange) or irrelevant HLA‐A*02:01/pMHC‐CMV‐pp65^NLV^ tetramer (black) stainings of CD8^neg^ T cells transduced with TCRs that were found (**D**) or that were not found ex *vivo* (**E**). *Shown are data from one representative experiment (performed twice) using PBMCs from one donor*. Abbreviations: MACS, Magnetic Activated Cell Sorting. irr.pMHC, irrelevant pMHC tetramer. wt, wild type. CDR, Complementary Determining Region. TRAV/TRBV, T‐cell Receptor Alpha/Beta Variable. TRBD, T‐cell Receptor Beta Diversity. TRAJ/TRBJ, T‐cell Receptor Alpha/Beta Joining. TRAC/TRBC, T‐cell Receptor Alpha/Beta Constant

Analysis of HLA‐A*02:01‐restricted EBV‐LMP2^FLY^‐specific TCR‐repertoires showed the presence in 9 out of 11 individuals of a public TCR‐CDR3β region [CASSYQGGNYGYTF] [16, 20]. T‐cell clones that were generated from two individuals that expressed this public CDR3β sequence or highly‐similar CDR3β‐sequences (CASSPQGGGDGYTF, CASSRQGGSYGYTF, CASSGQGGGDGYTF), revealed to express a public CDR3α [CATEGDSGYSTLTF] as well. Moreover, 10 highly similar receptors were also found, which only differed at position 5 of the CDR3β. Also, highly similar receptors were found to differ at position 9/10 of the CDR3β sequence, but all 10 highly‐similar receptors at least expressed the [NY] motif at positions 9/10. Since nine amino acids were never found at position 5 of the CDR3β, we asked whether these would interfere with the ability of the receptor to recognize EBV‐LMP2^FLY^ in HLA‐A*02:01. We first modeled the public TCR, with an [Y] on position 5 which was encoded by the germline TRBV6‐5 gene (from here on referred to as WT TCR), using a TCRmodeling algorithm to visualize position 5 in respect to HLA‐A*02:01 and the FLYALALLL peptide (Supplementary Figure 2) [21]. Although no formal crystal structure is known, position 5 seems to be at the spot whereby interaction with the peptide‐HLA‐complex cannot be ruled out, suggesting that the 9 highly similar CDR3β‐sequences that contained amino acids on position 5 that were not found could have resulted in a loss of specificity. To further test this, we generated a TCR panel of this public TCR in which we substituted the Tyrosine [Y] at position 5 of the CDR3β‐sequence with all 19 other amino acids. We distinguished the public WT CDR3β variant (Red) and 10 highly‐similar CDR3β variants that were found *ex vivo* (Blue) in healthy individuals from the 9 CDR3β variants that were not found *ex vivo* (Orange) (Figure 1A). All artificially generated EBV‐LMP2^FLY^‐specific TCRs were introduced into primary CD8^pos^ T cells isolated from peripheral blood of a healthy EBV‐seronegative, HLA‐A*02:01‐negative donor. Transduction efficiencies ranged from 6% to 43% (Figure S3A: left) and MACS enrichment, based on the presence of a murine‐TCR Cβ epitope present in the transgenic TCRs [22, 23], yielded populations in which 84–99% T cells expressed the transgenic TCRs (Figure S3A: right). Specific binding of HLA‐A*02:01/pMHC‐EBV‐LMP2^FLY^ tetramers was demonstrated for all EBV‐LMP2^FLY^‐specific TCR‐transduced CD8^pos^ T‐cell populations, regardless of whether the substituted amino acids were found (Figure 1B) or not found *ex vivo* (Figure 1C). Although all TCRs were able to bind EBV‐LMP2^FLY^‐specific pMHC‐tetramer, heterogeneous binding was observed for TCR 13 [C], TCR 16 [D], and TCR 17 [E] transduced CD8^pos^ T cells, and low binding was observed for TCR 18 [K]. TCR‐transduced CD8^pos^ T cells did not stain with a negative control pMHC‐tetramer containing an irrelevant HLA‐A*02:01‐restricted CMV peptide (Representative examples; Figure 1B and 1C; pMHC‐irr).

Heterogeneous EBV‐LMP2^FLY^‐specific pMHC‐tetramer binding could be indicative of low TCR avidity, with differences in functionality. To investigate the TCR avidity, we evaluated dependence on CD8 for binding to pMHC‐EBV‐LMP2^FLY^‐specific tetramers by introducing all 20 EBV‐LMP2^FLY^‐specific TCRs into primary CD4^pos^ T cells (CD8^neg^) isolated from a healthy EBV‐seronegative, HLA‐A*02:01‐negative donor. TCR‐transduction efficiencies ranged from 17–58% and further purification based on the expression of the introduced murine constant region resulted in pure TCR‐transduced CD8^neg^ T cells (range 90–99%; Figure S1B). Overall, a mean 1.9 (range 1.2–3.0) fold lower Mean Fluorescence Intensity (MFI) of EBV‐LMP2^FLY^‐specific tetramer binding was observed for TCRs transduced in CD8^neg^ T cells compared to CD8^pos^ T cells (Figure 1D, 1E and Figure S4a). Surface expression of the introduced TCRs was similar in CD8^neg^ and CD8^pos^ T cells, as determined by the MFI of the murine TCR‐Cβ (Figure S4b). However, CD8^neg^ T cells transduced with TCR 3 [G] and TCR 16 [D] showed low and heterogeneous EBV‐LMP2^FLY^‐specific pMHC‐tetramer staining. Because the endogenous TCR was still present in these TCR‐transduced T cells, competition for CD3 between the endogenous TCR and newly introduced TCR, could contribute to the heterogeneity in pMHC‐tetramer binding. Therefore, we additionally transduced all EBV‐LMP2^FLY^‐specific TCRs in CD8‐transduced Jurkat E6 cells that did not express an endogenous TCR (ΔTCR). Heterogeneous EBV‐LMP2^FLY^‐specific pMHC‐tetramer staining was not observed in CD8^pos^ or CD8^neg^ Jurkat E6 cells (Figure S5). Although CD8^neg^ Jurkat E6 cells transduced with TCR 3 [G] and TCR 16 [D] clearly bound EBV‐LMP2^FLY^‐specific pMHC‐tetramer, both pMHC‐tetramers stained with the lowest MFI. Overall, these data show that all TCRs were able to bind EBV‐LMP2^FLY^‐specific pMHC‐tetramers in CD8^pos^ T cells and to some extent when transduced into CD8^neg^ T cells. Importantly, while some amino acids (G and D) did diminish binding, this did not correlate with their prevalence in primary EBV‐LMP2^FLY^‐specific repertoires, where variants with a G residue on position 5 were found.

### Functionality of EBV‐LMP2^FLY^‐specific TCR‐transduced primary CD8^pos^ and CD8^neg^ T cells

**Figure 2:**
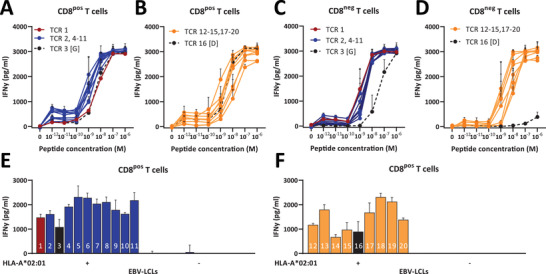
Functionality of EBV‐LMP2^FLY^‐specific TCR‐transduced primary CD8^pos^ and CD8^neg^ T cells. K562 cells were loaded with titrated concentrations of EBV‐LMP2^FLY^ peptide for 16 hours and were used to investigate recognition by TCR‐transduced primary CD8^pos^ and CD8^neg^ T cells. EBV‐transformed B lymphoblastoid cell lines (EBV‐LCLs) were used to study the recognition of endogenously produced EBV‐LMP2^FLY^ peptide.IFNγ was measured by standard ELISA. **A** and **B**) Shown are responses of TCR‐transduced CD8^pos^ T cells with TCRβ‐sequences that were found (**A**) or that were not found *ex vivo* (**B**). **C** and **D**) Shown are responses of TCR‐transduced CD4^pos^ (CD8^neg^) T cells with TCRβ‐sequences that were found (**C**) or that were not found *ex vivo* (**D**). **E** and **F**) IFNγ production upon stimulation with HLA‐A*02:01 positive EBV‐LCL is shown for TCR‐transduced CD8^pos^ T cells with TCRβ‐sequences that were found (**E**) or that were not found (**F**) *ex vivo*. HLA‐A*02:01 negative EBV‐LCLs were used as negative control. Black symbols with dotted lines and black bars indicate TCRs that performed poorly when transduced into CD4^pos^ (CD8^neg^) T cells. *Shown are data with means with standard deviations carried out in triplicate of one representative experiment (performed twice)*.

To investigate whether the functional reactivity of EBV‐LMP2^FLY^‐specific TCR‐transduced primary CD8^pos^ and CD8^neg^ T cells would be affected by the amino acid substitutions at position 5 of the CDR3β sequence, we performed a stimulation with HLA‐A*02:01‐transduced K562 cells exogenously loaded with varying concentrations of the EBV‐LMP2^FLY^ peptide. Similar dose‐dependent induction of IFNγ production was observed by CD8^pos^ T cells transduced with WT [Y] EBV‐LMP2^FLY^‐specific TCRs or all other 19 TCR variants with only minor differences in sensitivity to peptide dose (Figure 2A and 2B). Moreover, there was no clear difference in the sensitivity to peptide dose between TCR variants that were found *ex vivo* and those that were not. In fact, some of the latter even exhibited greater sensitivity than the WT [Y] TCR eliciting production of IFN‐γ at lower peptide concentrations (Figure 2A and 2B). Two TCR variants, TCR 3 [G] and TCR 16 [D], were less able to elicit production of IFN‐γ when expressed in CD8^neg^ T cells. TCR 3 [G] was only able to produce IFNγ at high peptide concentrations, while TCR 16 [D] seemed to be non‐functional (Figure 2C and 2D). This reduced functionality of TCR 3 [G] and TCR 16 [D] was also observed when expressed in CD8^neg^, but not in CD8^pos^ Jurkat E6^ΔTCR^ cells, as measured by upregulation of CD69 (Figure S6). The limited functionality of TCR 3 [G] and TCR 16 [D] was in line with previous EBV‐LMP2^FLY^‐specific pMHC‐tetramer staining results. To investigate whether EBV‐LMP2^FLY^‐specific TCR‐transduced primary CD8^pos^ T cells were also capable of recognizing endogenously processed LMP2^FLY^, we performed a stimulation with an HLA‐A*02:01^pos^ EBV‐transformed lymphoblastoid cell line (EBV‐LCL). Similar induction of IFNγ production upon stimulation with the HLA‐A*02:01^pos^ EBV‐LCL was observed for CD8^pos^ T cells with EBV‐LMP2^FLY^‐specific TCRs found *ex vivo* (Figure 2E) and with EBV‐LMP2^FLY^‐specific TCRs not found *ex vivo* (Figure 2F). No IFNγ production was observed upon stimulation with HLA‐A*02:01 negative EBV‐LCLs. In conclusion, there was no correlation between the presence or absence of specific TCR variants in the naturally occurring *ex vivo* HLA‐A*02:01‐restricted EBV‐LMP2^FLY^‐specific TCR‐repertoire and functionality. Although two TCR variants exhibited limited pMHC‐tetramer staining and reduced functionality, especially in a CD8^neg^ context, one of these was found in the *ex vivo* repertoire, while the other was not found in fluorescence activated cell sorted (FACS) enriched and *in vitro* expanded EBV‐LMP2^FLY^‐specific T‐cell populations of the 11 individuals that were studied. This might suggest that these properties in CD8^neg^ T cells are not factors important for natural selection. Most importantly, all artificially generated TCR variants showed equal functionality in the presence of CD8, which is their natural context. We therefore conclude that the absence of specific amino acids on position 5 in the *ex vivo* found HLA‐A*02:01‐restricted EBV‐LMP2^FLY^‐specific “family” of public TCRs is not explained by constraints on TCR binding to the EBV‐LMP2^FLY^‐ HLA‐A*02:01 complex.

### Occurrence of amino acids on position 5 of the CDR3β correlate with the recombination probability

**Figure 3:**
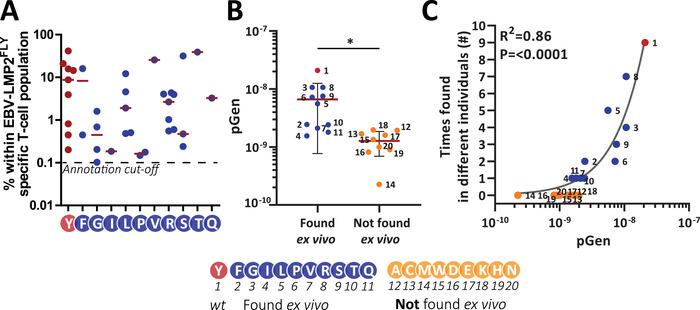
Optimized Likelihood estimate of immunoGlobulin Amino acid sequences (OLGA) shows that the variants containing CDR3β sequences that were not found *ex vivo* have a lower recombination probability. **A)** Shown are frequencies of EBV‐LMP2^FLY^‐specific TCRβ‐sequences within each FACS enriched and expanded EBV‐LMP2^FLY^‐specific T‐cell population. Each dot represents a different individual. Only TCRβ‐sequences above 0.1% were used. **B**) Shown are the generation probabilities (pGen) of EBV‐LMP2^FLY^‐specific TCRβ‐sequences that were found (Red and Blue) or that were not found *ex vivo* (Orange). **C**) The EBV‐LMP2^FLY^‐specific TCRβ‐sequences were identified from a total of 11 healthy donors. We investigated if the generation probabilities (pGen) correlated with the number of times that these sequences were found in peripheral blood of 11 different donors. Statistical differences were assessed with the unpaired *t* test (**B**) and the Spearman's correlation coefficient *r* (**C**). Shown are the means (red lines) with standard deviations (error bars). *P<.05. Abbreviations: pGen, Recombination probability.

Despite the fact that the CDR3β sequence allows complete freedom to all amino acids on position 5, 9 amino acids were never found among a total of 251 different TCRs analyzed [16, 20], even though most of the other 11 amino acids were found repeatedly in FACS‐enriched and *in vitro* expanded EBV‐LMP2^FLY^‐specific T‐cell populations from different individuals, at frequencies ranging from 0.1%‐42% (Figure 3A). We reasoned that the absence of these amino acids might be a consequence of a bias in the recombination process [24, 25]. To test this idea, we computed the recombination probability (pGen) of all 20 possible CDR3β‐sequence variants. The recombination probability was calculated using the Optimized Likelihood estimate of Ig Amino acid sequences (OLGA) algorithm, which is able to compute the generation probabilities of TCR amino acid sequences [26]. The public WT CDR3β‐sequence (Red) and the 10 variants (Blue) that were found *ex vivo*, were significantly more likely to be generated than the variants containing one of the other 9 amino acids at position 5 (Orange) (Figure 3B; p = 0.014; unpaired *t* test, two‐tailed). Furthermore, the high recombination probability correlated with the number of individuals with EBV‐LMP2^FLY^‐specific T‐cell populations that contained these CDR3β‐sequences (Figure 3C). Therefore, we conclude that the bias against specific amino acid usage on position 5 of the CDR3β is most likely explained by constraints imposed by the recombination process.

## Discussion

In this study, we investigated whether all amino acids were possible at the highly‐variable position 5 of the CDR3β sequence of a public HLA‐A*02:01‐restricted EBV‐LMP2^FLY^‐specific TCR, independent of the amino acid properties (e.g. charge, hydrophobicity, and size). Position 5 of this CDR3β sequence [CASSxQGGNYGYTF], was recently identified as highly variable since 11 different, but highly‐similar TCRs, were found *ex vivo* in EBV‐LMP2^FLY^‐specific T‐cell populations from healthy individuals [16]. It was reported before that TCRs with a cysteine [C] in the CDR3β sequence would be dysfunctional and CDR3β sequences with positively charged and hydrophobic amino acids are disfavored [11]. However, we demonstrated that the amino acids on position 5 of the EBV‐LMP2^FLY^‐specific CDR3β sequence were completely interchangeable and they did not significantly influence the specificity or functionality as long as CD8 was present. Heterogeneous pMHC‐tetramer staining was observed in TCR‐transduced CD8^pos^ T cells, but not when these TCRs were transduced in Jurkat E6 cells, showing that the heterogeneous staining was not due to intrinsically insufficient pairing of the artificial TCRα and TCRβ‐chains. Since there was no clear difference in functionality between variants, it is likely that certain CDR3β sequences were not found *ex vivo* in EBV‐LMP2^FLY^‐specific T‐cell populations due to limitations in the genetic recombination process. We indeed showed that CDR3β sequences that encode for the “missing” 9 amino acids had a lower chance to be generated during V‐D‐J recombination (recombination probability [26]) compared to the 11 TCRβ sequences that were found in EBV‐LMP2^FLY^‐specific T‐cell populations *ex vivo*.

We and others have found many TCRs that are highly similar to public TCRs and these highly‐similar TCRs often differed on specific positions in the CDR3 region [13‐16, 20, 22, 27]. Position 5 of our public TCR [CASSYQGGNYGYTF] was not the only position that differed on a specific position compared to other highly‐similar TCRs. Also position 9/10 [NY] showed high‐variability, but not as much as position 5. Because the majority of different amino acids were found on position 5 in combination with the [NY] sequence, we kept this part constant for all artificially generated TCRs.Whether only specific amino acids are allowed on such variable positions in the CDR3 regions or whether such positions are completely interchangeable with any amino acid had not yet been investigated. It has been described that some CDR3 sequences have a higher likelihood to be generated by V(D)J‐recombination, also known as the recombination probability [26, 28]. Therefore, based on chance, some amino acids could be less frequently observed in the CDR3‐regions that could explain why not every variant was found. Indeed, for our public WT TCR, the first 5 amino acids are directly derived from the TRBV6‐5 gene and the last 6 amino acids are from the TRBJ1‐2 gene without modifications, explaining why this variant has the highest generation probability and occurrence *in vivo*. Other variants that were found require additional trimming of nucleotides encoding the tyrosine [Y] at the end of the TRBV6‐5‐gene. Therefore, based on chance, some variants might be more likely to be found. Increasing the number of individuals will most likely ultimately result in the identification of more TCR variants from the “not found” group of TCRs. From a TCR point of view to dock to the peptide‐HLA complex, it may be assumed that the CDR3β‐sequence only allows substitutions of amino acids with similar propertiesor that position 5 does not interfere/interact with the peptide‐HLA complex. However, the TCRmodel [21] used to visualize the TCR‐peptide‐HLA structure showed that positions 5, 6 and 7 are at the loop of the CDR3β region, which is known to interact with the peptide‐HLA. However, important to note, this is not a formal crystal structure, but a model generated using the TCR model algorithm [21]. For the WT tyrosine [Y] amino acid, we expected to find highly similar TCRs with amino acids on position 5 with the same polarity [S, T, N, and Q] or with the same aromatic structure [F]. However, positively charged [R] and non‐polar [G, V, P, L, and F] amino acids were also found *ex vivo*. There was no correlation in charge, size or polarity of the amino acids that were found *ex vivo* compared to those that were not. More strikingly, the hydrophobic amino acid tyrosine [Y] is very similar to phenylalanine [F] since they both contain an aromatic ring and are only differing by an ‐OH group. However, T cells expressing EBV‐LMP2^FLY^‐specific TCRs with an [F] on position 5 of the CDR3β sequence were only found in 2 individuals, while T cells expressing TCRs with a [Y] on position 5 were found in 9 individuals, which correlates with the recombination probability, but not with the similarity of the amino acids. It has also been recently demonstrated that the CDR3α and even CDR1α contribute strongly to the peptide specificity [29, 30]. It could be argued that minor alterations in the CDR3β are allowed as long as sufficient binding to the peptide‐HLA is maintained by other CDR regions, thus belonging to one public TCR family.

Lower tetramer staining and functionality as observed for certain amino acids in TCR‐transduced CD8^neg^ T cells may indicate differences in TCR affinity, but this apparently is insufficient to result in reduced functional avidity of CD8^pos^ T cells expressing these TCRs. Stability of the TCR‐CD4/CD8 coreceptor‐MHC interactions and half‐lives of the TCR‐MHC complex are more likely to play a role in the differences observed. It is already known that CD8 can stabilize peptide/MHC‐class‐I binding of TCRs [31]. In contrast, past studies and recent *in situ* measurements at intercellular junctions, show that CD4 does not stabilize interactions of TCRs with their natural peptide/MHC‐class‐II molecules [32, 33] This makes it unlikely that CD4 can stabilize interactions of TCRs with peptide/MHC‐class‐I molecules, although this has not been tested. Some of our generated TCR variants might therefore have a shorter half‐life in CD4 T cells resulting in more heterogeneous/lower tetramer staining.

However, other rules may apply to the CDR3‐region. It has recently been shown that positively charged and hydrophobic amino acids in CDR3β sequences are disfavoured and TCRs with cysteines in their CDR3 peptide‐binding regions are clonally deleted [11]. Although these amino acids were mentioned to be disfavoured, the majority of the hydrophobic amino acids and the positively charged amino acid arginine [R] were found in the CDR3‐regions of EBV‐LMP2^FLY^‐specific TCRs. Interestingly, cysteines were found in the CDR3 regions of thymocytes that did not undergo thymic selection yet [11, 34]. Why cysteines are not found in CDR3 regions of matured T cells is not completely understood, but we show that positively charged, all hydrophobic amino acids and cysteine on position 5 of the CDR3β region of the EBV‐LMP2^FLY^‐specific TCR did not impair the specificity and functionality. It might be that thymocytes with cysteines were negatively selected during thymic development because of a too strong TCR‐signaling [34], which prevents maturation into autoreactive T cells. However, we did not find any autoreactivity of TCR‐transduced T cells when stimulated with K562 cells that were transduced with HLA‐A*02:01 when no peptide was added.

Our data show that the amino acids on position 5 of the EBV‐LMP2^FLY^‐specific CDR3β sequence, were completely inter‐changeable in CD8 T cells and did not significantly alter the specificity and functionality. We show that the recombination probability drives the possibility to find all possible amino acids on this position. This data implies that TCRs that are highly similar to the public TCR, and differ in amino acids on such promiscuous positions in the CDR3β, should be considered as one public TCR family.

## Materials and methods

### Cell collection and culturing conditions

EBV‐LMP2‐FLYALALLL (LMP2^FLY^)‐specific T‐cell populations were isolated from peripheral blood mononuclear cells (PBMCs) from 11 healthy donors as described previously [35]. In short, EBV‐LMP2^FLY^‐specific T‐cell populations were enriched by fluorescently activated cell sorted (FACS) using EBV‐LMP2^FLY^‐specific peptide‐HLA complexes. EBV‐LMP2^FLY^‐specific T‐cell populations were non‐specifically expanded and purity was checked after 2 weeks of culture [35]. Healthy donors (HLA‐A*02:01^neg^ and EBV^neg^) were selected to isolate primary CD4^pos^ and C(((D8^pos^ T cells for transduction of the artificially generated TCR‐sequences. These primary CD4^pos^ and CD8^pos^ T cells were isolated using magnetic activated cell sorting MACS) with CD4 and CD8 T‐cell isolation kits with LS columns from Miltenyi Biotec (Bergisch Gladbach, Germany). Additional CD25‐beads (Miltenyi) were added during CD4^pos^ T‐cell isolation to deplete regulatory T cells. All primary T cells were cultured in Iscove's Modified Dulbecco's Medium (IMDM; Lonza, Basel, Switserland) additionally containing 5% heat‐inactivated human serum (ABOS; Sanquin reagents, Amsterdam, The Netherlands), 5% heat‐inactivated fetal bovine serum (FBS; Invitrogen, Carlsbad, USA), 2.7 mM L‐glutamine Lonza), 100U/mL Penicillin (Lonza), 100 μg/mL streptavidin (Lonza) and 100 IU/mL recombinant‐Interleukin‐2 (IL‐2; Chiron, Emeryville, USA), otherwise referred to as T‐cell medium (TCM). EBV‐LMP2^FLY^‐specific T‐cell populations and primary T cells transduced with artificially generated TCRs were expanded using TCM supplemented with 0.8 μg/mL phytohemagglutinin (PHA; Oxoid Limited, Basingstoke, UK) and five‐fold 35gy irradiated autologous or allogeneic PBMCs as feeder cells. The endogenous TCR of Jurkat E6 (Clone E6‐1 ATCC^®^ TIB‐152) cells was knocked‐out (∆TCR) using a previously described approach [22, 36] and in specific experiments Jurkat E6 ∆TCR cells were transduced with CD8alpha/CD8beta LZRS‐plasmid). EBV‐transformed lymphoblastic cell lines (EBV‐LCLS) were generated according to standard protocols [37]. EBV‐LCLs, Jurkat E6, and K562 (ATCC^®^ CCL‐243) cells were cultured in stimulator medium consisting of IMDM (Lonza) supplemented with 10% FBS (Invitrogen), 100U/mL penicillin (Lonza), 100 μg/mL streptavidin (Lonza), and 2.7 mM L‐glutamine (Lonza).

### Generation of peptide‐HLA‐A*02:01 complexes

CMV‐pp65‐NLVPMVATV(pp65^NLV^) and EBV‐LMP2^FLY^ peptides were synthesized in‐house using standard Fmoc chemistry. Recombinant HLA‐A*02:01 heavy chain and human β2m light chain were in‐house produced in Escherichia coli. MHC‐class‐I refolding was performed as previously described with minor modifications [38]. MHC‐class‐I complexes were purified by gel‐filtration using fast protein liquid chromatography (FPLC). Peptide‐MHC (pMHC) tetramers EBV‐LMP2^FLY^/HLA‐A*02:01 and CMV‐pp65^NLV^/HLA‐A*02:01 were generated by labeling of biotinylated pMHC‐monomers with streptavidin‐coupled phycoerythrin (PE; Invitrogen, Carlsbad, USA). Complexes were stored at −80 °C. Formation of stable pMHC‐monomers was assessed using UVexchange technology [39] according to a previously described protocol [40].

### Retroviral transductions and enrichments

The construct encoding the HLA‐A*02:01 sequence was coupled to an IRES sequence with a truncated form of the nerve growth factor receptor (tNGFR) and was cloned into an LZRS plasmid. This construct was verified using reverse transcriptase polymerase chain reactions (RT‐PCR) and Sanger sequencing. As an additional control, tNFGR only was cloned into an LZRS plasmid (mock). Retroviral transduction was performed as previously described [41]. K562 WT cell lines were transferred to wells containing stable retroviral particles, generated using a puromycin‐selected stable ϕ‐NX‐amphotropic packaging cell line, and incubated for 24 h at 37°C [42]. Transduced cell lines were subsequently enriched by Fluorescent Activated Cell Sorting (FACS) for expression of tNGFR using APC‐labeled tNGFR antibodies (CD271; Southern Biotech Associations, Alabama, USA). In total, a median of 1 × 10^6^ cells (range 0.4‐6 × 10^6^) FACS‐enriched EBV‐LMP2^FLY^‐specific T cells were used to determine the TCR variable beta(β) sequence. TCR variable beta(β) sequences used by FACS‐enriched EBV‐LMP2^FLY^‐specific T‐cell populations were determined using ARTISAN PCR adapted for TCR PCR as previously described [16, 22, 43]. CDR3β‐sequences were built using MIXCR software with [default] settings and a limit of processing 10 × 10^6^ input sequences per FACS enriched EBV‐LMP2^FLY^‐specific T‐cell population in combination with a bi‐directional reading ((approach 5’‐3’ and 3’‐5’ read)16, 44). As a cut‐off, all sequences present above 0.1% in the enriched EBV‐LMP2^FLY^‐specific T‐cell populations were included in the analyses [16]. T‐cell clones were generated from EBV‐LMP2^FLY^‐specific T‐cell populations using limited dilution to determine the TCR variable alpha(α) sequences using SANGER sequencing. Twenty different retroviral vectors that contained the codon‐optimized cys–cys TCR‐constant‐modified T‐cell receptor α sequence: TRAV17, CDR3α [CATEGDSGYSTLTF] TRAJ11, and cys–cys TCR‐constant‐modified varying β sequence: TRBV6‐5, CDR3β [CASSxQGGNYGYTF] TRBJ1‐2 with all 20 different amino acids on position 5 were constructed on MP71 backbones with murineTCR constant (mTCR‐C) α/β sequences and joined by a P2A sequence as previously described [18, 22] and ordered from Baseclear (Leiden, The Netherlands). Codon‐optimization was for all TCR‐constructs the same, and only differed on position 5 of the CDR3 β sequence (Figure S1). ϕ‐NX‐amphotropic packaging cells were transfected with MP71 vectors and pCL‐ampho retrovirus packaging using FuGENE HD (Roche, Basel, Switzerland) according to the manufacturer's instructions and retroviral supernatant was harvested after 48 hours. Primary HLA‐A*02:01^neg^ and EBV^neg^ CD4^pos^ and CD8^pos^ T cells were non‐specifically activated for 48 h using an autologous feeder mixture and PHA as described above, prior transduction. Primary T cells and Jurkat E6 cells were transduced with retroviral supernatant that contained the TCRα and TCRβ sequences in rectronectin‐coated 24 wells‐plates (100,000 cells per well). To determine the purity of MACS‐isolated primary CD8^pos^ and CD4^pos^ T cells, the cells were stained with PE‐conjugated anti‐CD4 or anti‐CD8 (BD Pharmingen) for 20 min at 4 °C. MACS enrichments using APC‐labeled mTCR‐Cβ antibodies (BD) and anti‐APC‐microbeads (Miltenyi) were performed in order to purify TCR‐transduced populations. Transduction efficiencies and purities after MACS enrichments were assessed after 10 days of culturing by staining transduced cells with APC‐labeled mTCR‐Cβ‐specific antibodies (BD) for 20 min at 4°C. Prior to mTCR‐Cβ staining, cells were stained with PE‐labeled HLA‐A*02:01/pMHC‐LMP2^FLY^‐specific pMHC‐tetramers to determine their capacity to bind HLA‐A*02:01/pMHC‐LMP2^FLY^‐specific pMHC‐tetramers. As a control, cells were stained with PE‐labeled HLA‐A*02:01/pMHC‐CMV‐pp65^NLV^‐specific pMHC‐tetramers.

### Functional assays

IFN‐γ production by TCR‐transduced primary T cells was quantified using standard enzyme‐linked immunosorbent assays (ELISA) according to the manufacturer's instructions (Diaclone, Besançon, France). Responder T cells were co‐cultured with stimulator cells at a ratio of 1:5 (responder: stimulator) for 16 h at 37°C in TCM using 25 IU/mL IL‐2 instead of 100 IU/mL IL‐2. To measure activation of TCR‐transduced Jurkat E6^∆TCR^ cell‐lines upregulation of activation marker CD69 was analyzed using flow cytometry. Responder TCR‐transduced Jurkat E6^∆TCR^ cells were stimulated with HLA‐A*02:01‐transduced K562 cell‐lines with or without exogenous peptide loading (10^−6^ M) at a ratio of 1:10 (responder:stimulator, R:S) in stimulator medium for 16 h at 37°C. After O/N incubation, cells were washed twice before adding CD69‐PE (Invitrogen), mTCR‐Cβ‐APC (BD), and CD8‐PerCP (BD) monoclonal antibodies for 30 min at 4°C. All analyses were performed on a FACS Calibur (BD), and analyzed using Flowjo Software (TreeStar, Ashland, USA).

## Conflict of interest

The authors declare no commercial or financial conflict of interest.

## Ethics approval

After informed consent according to the Declaration of Helsinki, healthy individuals (homozygously) expressing HLA‐A*02:01 and HLA‐B*07:02 were selected from the Sanquin database and the biobank of the department of Hematology, Leiden University Medical Center (LUMC).

## Authorship

W.H., M.G., D.A.T.L., and L.H. performed experiments, A.S.S. designed and ran the R‐script for the OLGA algorithm, W.H. analyzed results and made the figures, W.H., J.H.F.F., D.A. and I.J. designed the research and wrote the paper.

### Peer review

The peer review history for this article is available at https://publons.com/publon/10.1002/eji.202249975

**Abbreviations**
CDRs: complementary determining regions
EBV: Epstein Barr virus
EBV‐LCL: EBV‐transformed lymphoblastoid cell line
FACS: Fluorescent activated cell sorting
FLY peptide: FLYALALLL peptide
HLA: human leukocyte antigen
LMP2: latent memrane protein 2
MACS: Magnetic activated cell sorting
MFI: Mean fluorescence intensity
pGen: recombination probability
TCR: T‐cell receptor
VDJ genes: Variable Diversity Joining genes

## Supporting information

Supplementary Figure 1. Codon optimization of EBV‐LMP2^FLY^ ‐specific TCRs.
Supplementary Figure 2. TCR‐pHLA *model* of EBV‐LMP2^FLY^ ‐specific TCR expressing CDR3β‐CASSYQGGNYGYTF.
Supplementary Figure 3. Transduction efficiency of EBV‐LMP2^FLY^ ‐specific TCRs and purity of enriched TCR‐transduced primary T cells
Supplementary Figure 4. Introduced EBV‐LMP2^FLY^ ‐specificity in primary T cells
Supplementary Figure 5. Amino acid substitutions in the complementary determining region 3 of HLA‐A*02:01‐restricted EBV‐LMP2‐specific TCRs show overall maintenance of specificity in Jurkat E6 cells
Supplementary Figure 6. TCR gene transfer introduced EBV‐LMP2^FLY^ specificity and reactivity into Jurkat E6 cells

## Data Availability

The data that support the findings of this study are openly available on Mendeley at DOI: 10.17632/zzyrgzcwdp.1, (https://data.mendeley.com/datasets/zzyrgzcwdp/1)
