# Supplementary material for: Amino acids at position 5 in the peptide/MHC binding region of a public virus‐specific TCR are completely inter‐changeable without loss of function
Source: Eur J Immunol. 2022 Oct 19;52(11):1819–28. doi: 10.1002/eji.202249975 (PMC9828479; doi:10.1002/eji.202249975)
Supplement: Supplementary file 1 — Supplementary Figure 1. Codon optimization of EBV‐LMP2FLY ‐specific TCRs. Supplementary Figure 2. TCR‐pHLA model of EBV‐LMP2FLY ‐specific TCR expressing CDR3β‐CASSYQGGNYGYTF. Supplementary Figure 3. Transduction efficiency of EBV‐LMP2FLY ‐specific TCRs and purity of enriched TCR‐transduced primary T cells Supplementary Figure 4. Introduced EBV‐LMP2FLY ‐specificity in primary T cells Supplementary Figure 5. Amino acid substitutions in the complementary determining region 3 of HLA‐A*02:01‐restricted EBV‐LMP2‐specific TCRs show overall maintenance of specificity in Jurkat E6 cells Supplementary Figure 6. TCR gene transfer introduced EBV‐LMP2FLY specificity and reactivity into Jurkat E6 cells [file EJI-52-1819-s001.pdf]

Amino-acids at position 5 in the peptide/MHC binding region of a public virus-specific TCR are completely inter-changeable without loss of function

Wesley Huisman[1,2], Melanie de Gier[1], Lois Hageman[1], Alina S. Shomuradova[3], Didier A.T. Leboux.[1], Derk Amsen[2], J.H. Frederik Falkenburg J.H.F[1], Inge Jedema[1]

1 Department of Hematology, Leiden University Medical Center, The Netherlands
2 Department of Hematopoiesis, Sanquin Research and Landsteiner Laboratory for Blood Cell Research, Amsterdam, The Netherlands
3 Laboratory for Transplantation Immunology, National Research Center for Hematology, Moscow, Russia

Online Supplementary Appendix

Supplementary Figures

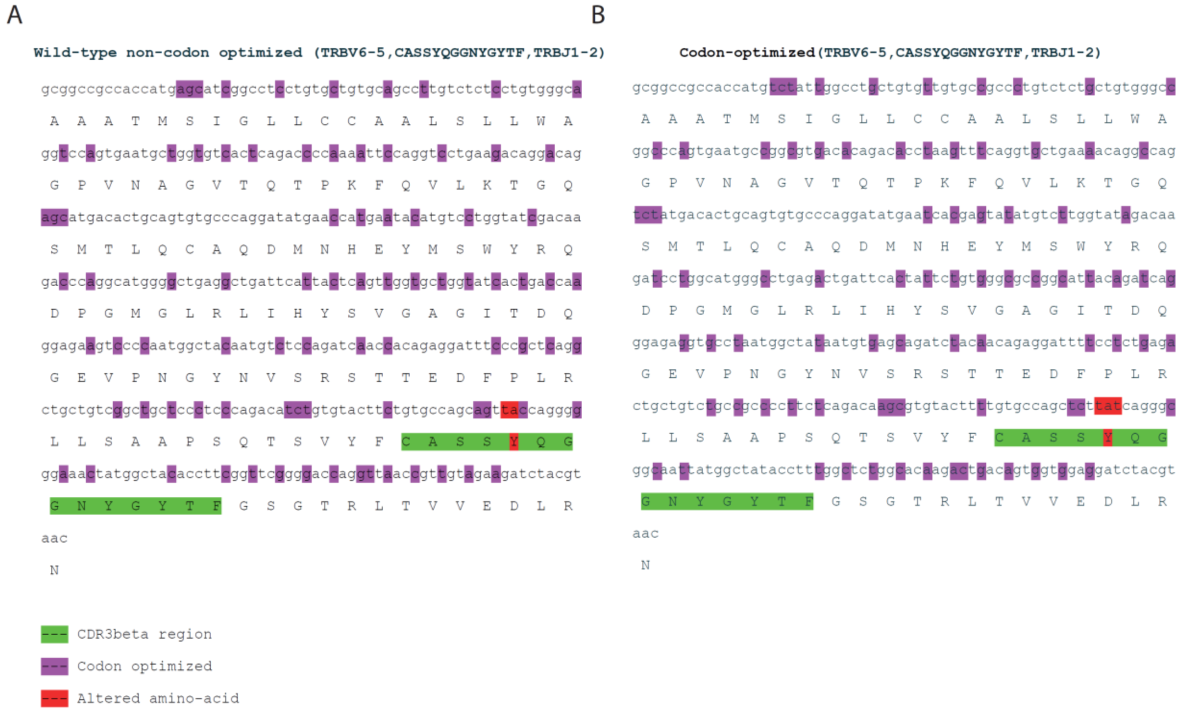

Supplementary Figure 1. Codon optimization of EBV-LMP2^{FLY}-specific TCRs. A) wild-type sequence of TCRβ (TRBV6-5-CASSYQGGNYGYTF-TRBJ1-2) expressing EBV-LMP2^{FLY}-specific T cells. B) codon-optimization of the CDR3β-CASSYQGGNYGYTF expressing EBV-LMP2^{FLY}-specific TCR. All other variants were made using this template and only the 3 red nucleotides were altered.

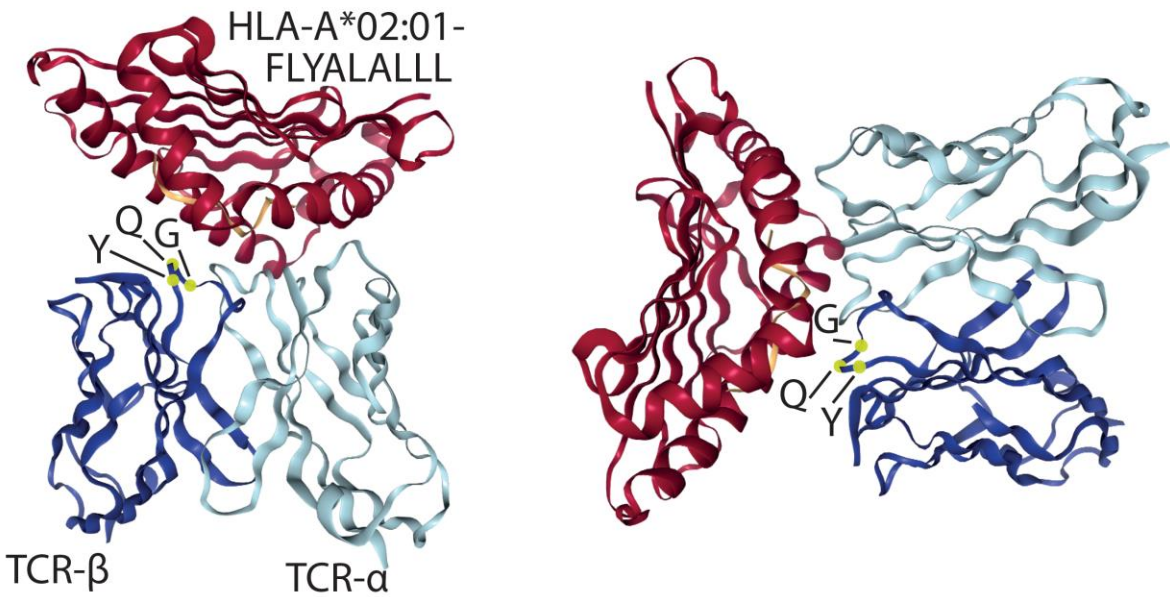

Supplementary Figure 2. TCR-pHLA model of EBV-LMP2^{FLY}-specific TCR expressing CDR3β-CASSYQGGNYGYTF. TCRmodel (https://tcrmodel.ibbr.umd.edu/) was used to generate the TCR-pHLA structure. The yellow dots represent positions 5 [Y], 6 [Q] and 7 [G] of the EBV-LMP2^{FLY}-specific TCR expressing CDR3β-CASSYQGGNYGYTF.

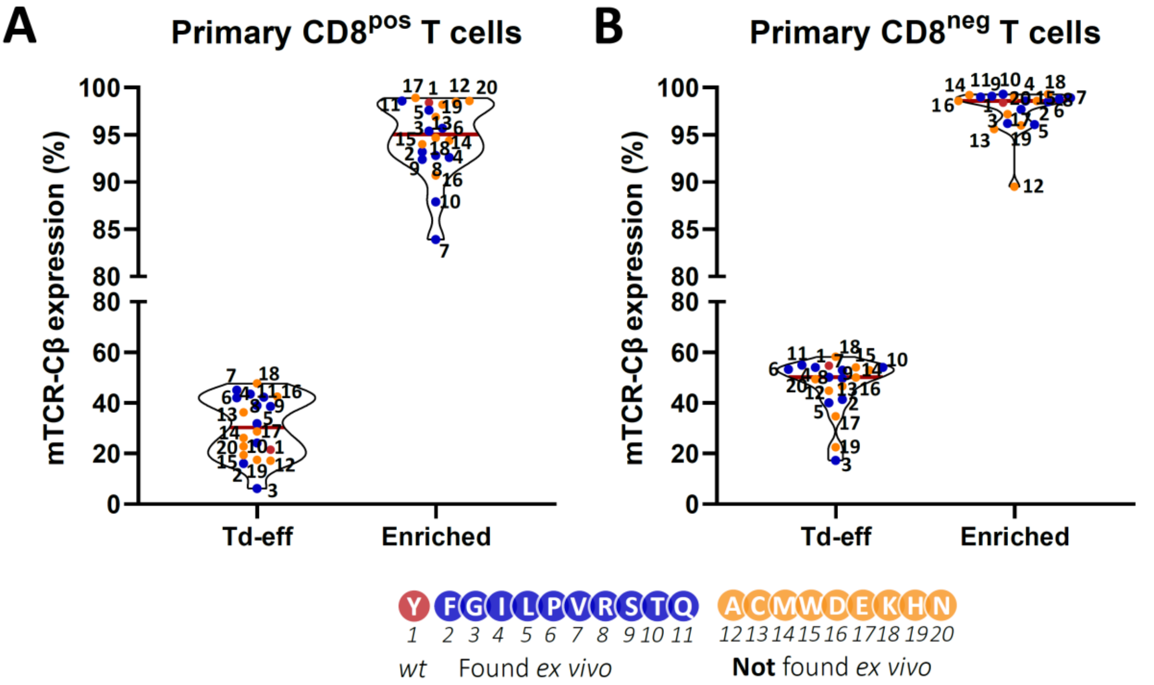

Supplementary Figure 3. Transduction efficiency of EBV-LMP2^{FLY}-specific TCRs and purity of enriched TCR-transduced primary T cells. Primary CD8^{pos} and CD4^{pos} (CD8^{neg}) T cells were isolated using Magnetic Activated Cell Sorting (MACS). Transduced primary cells were purified based on expression of murine-TCR-Cβ using MACS and expanded**.** The wildtype EBV-LMP2^{FLY}-specific TCRαβ-sequence is shown in red, additional TCRβ-sequences found *ex vivo* are shown in blue. TCRβ-sequences not found *ex vivo* are shown in orange. **A and B**) Shown is the transduction efficiency and purity after MACS enrichment of EBV-LMP2^{FLY}-specific TCR-transduced CD8^{pos} (**A**) and CD8^{neg} T cells (**B**).

Shown are medians (red) in violin plots with all individual samples.

Abbreviations: td-eff; transduction efficiency, wt; wildtype, mTCR-Cβ; murine TCR-constant β

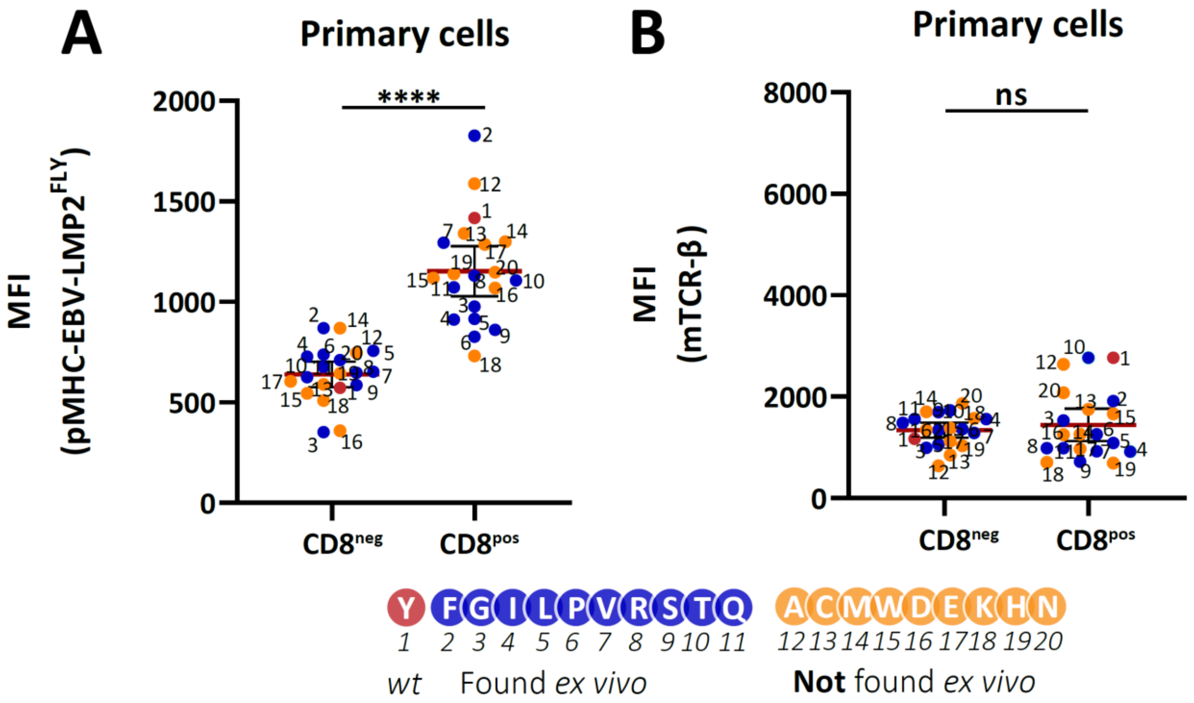

Supplementary Figure 4. Introduced EBV-LMP2^{FLY}-specificity in primary T cells. Primary CD4^{pos} (CD8^{neg}) and CD8^{pos} T cells were isolated using Magnetic Activated Cell Sorting (MACS). Transduced primary cells were purified based on expression of murine-TCR-Cβ using MACS and expanded**.** The wildtype public EBV-LMP2^{FLY}-specific TCRαβ-sequence is shown in red, additional highly-similar TCRβ-sequences found *ex vivo* are shown in blue. TCRβ-sequences not found *ex vivo* are shown in orange **A)** Mean Fluorescence Intensity (MFI) of pMHC-EBV-LMP2^{FLY} tetramer binding was assessed for CD8^{neg} and CD8^{pos} T cells. **B**) Shown is the MFI of the introduced TCR in CD8^{neg} and CD8^{pos} T cells as measured by expression of murine-TCR-Cβ.

Statistical differences were assessed with the paired *t* test (**A and B**).

Shown are means (red line) with 95% confidence intervals (error bars). ****P<.0001; ns; not significant >.05.

Abbreviations; MFI; Mean Fluorescence Intensity, Td; Transduced, wt; wildtype

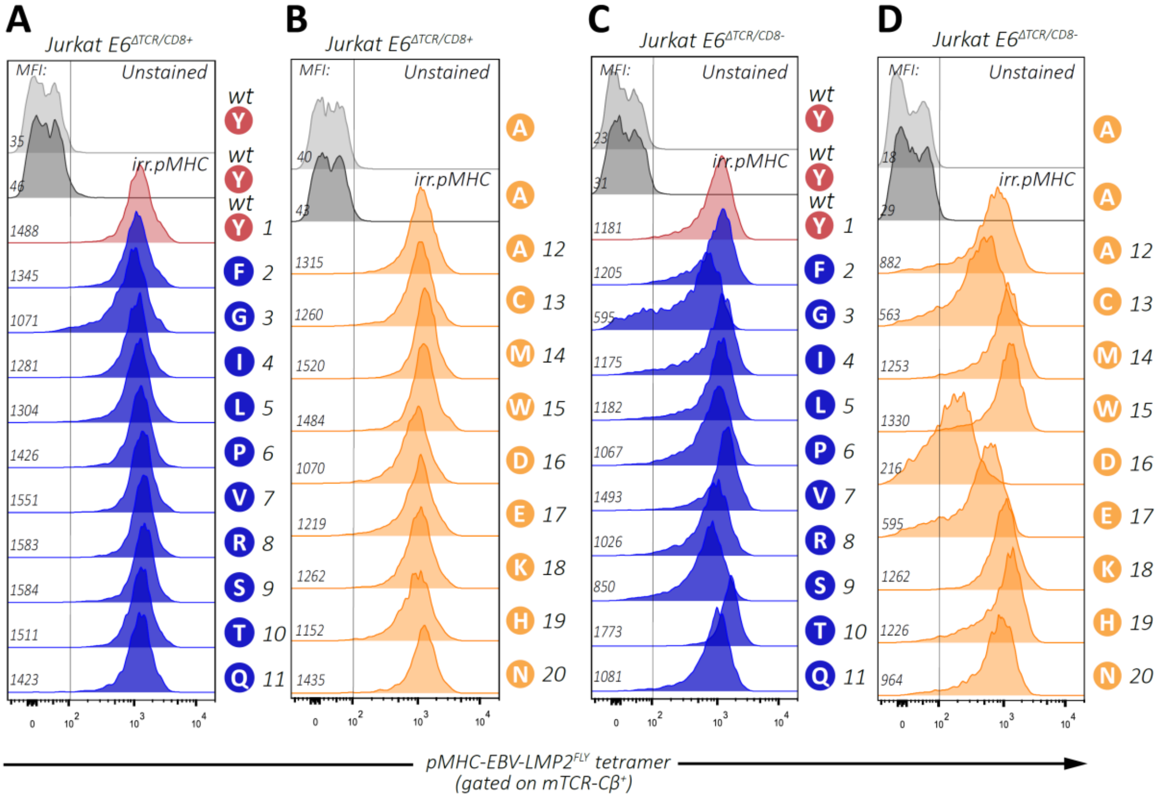

Supplementary Figure 5. Amino-acid substitutions in the complementary determining region 3 of HLA-A*02:01-restricted EBV-LMP2-specific TCRs show overall maintenance of specificity in Jurkat E6 cells. The endogenous TCRs of Jurkat E6 cells was knocked out using Crispr-Cas9 technology (ΔTCR). Jurkat E6^{ΔTCR} cells were transduced with CD8 to simulate CD8^{pos} T cells (Jurkat E6$^{ΔTCR/CD8+}$). Transduced cells were purified based on expression of murine-TCR-Cβ using MACS and expanded**.** Twenty different TCR-constructs were designed with amino-acids substitutions at position 5 of the CDR3β-sequence. The wildtype EBV-LMP2^{FLY}-specific TCRαβ-sequence is shown in red, additional highly-similar TCRβ-sequences found *ex vivo* are shown in blue. TCRs 12-20 were not found *ex vivo* and are shown in orange. **A and B)**. Shown are histograms of specific HLA-A*02:01/pMHC-EBV-LMP2^{FLY} tetramer (red, blue and orange) or irrelevant HLA-A*02:01/pMHC-CMV-pp65^{NLV} tetramer (black) stainings of Jurkat E6$^{ΔTCR/CD8+}$ cells transduced with TCRs that were found *ex vivo* (**A**) or that were not found *ex vivo* (**B**)*.* **C** and **D**) Shown are histograms of specific HLA-A*02:01/pMHC-EBV-LMP2^{FLY} tetramer (red, blue and orange) or irrelevant HLA-A*02:01/pMHC-CMV-pp65^{NLV} tetramer (black) stainings of Jurkat E6$^{ΔTCR/CD8-}$ cells transduced with TCRs that were found ex *vivo* (**C**) or that were not found ex *vivo* (**D**).

Abbreviations: irr.pMHC, irrelevant pMHC tetramer, MFI; Mean Fluorescent Intensity, wt; wildtype

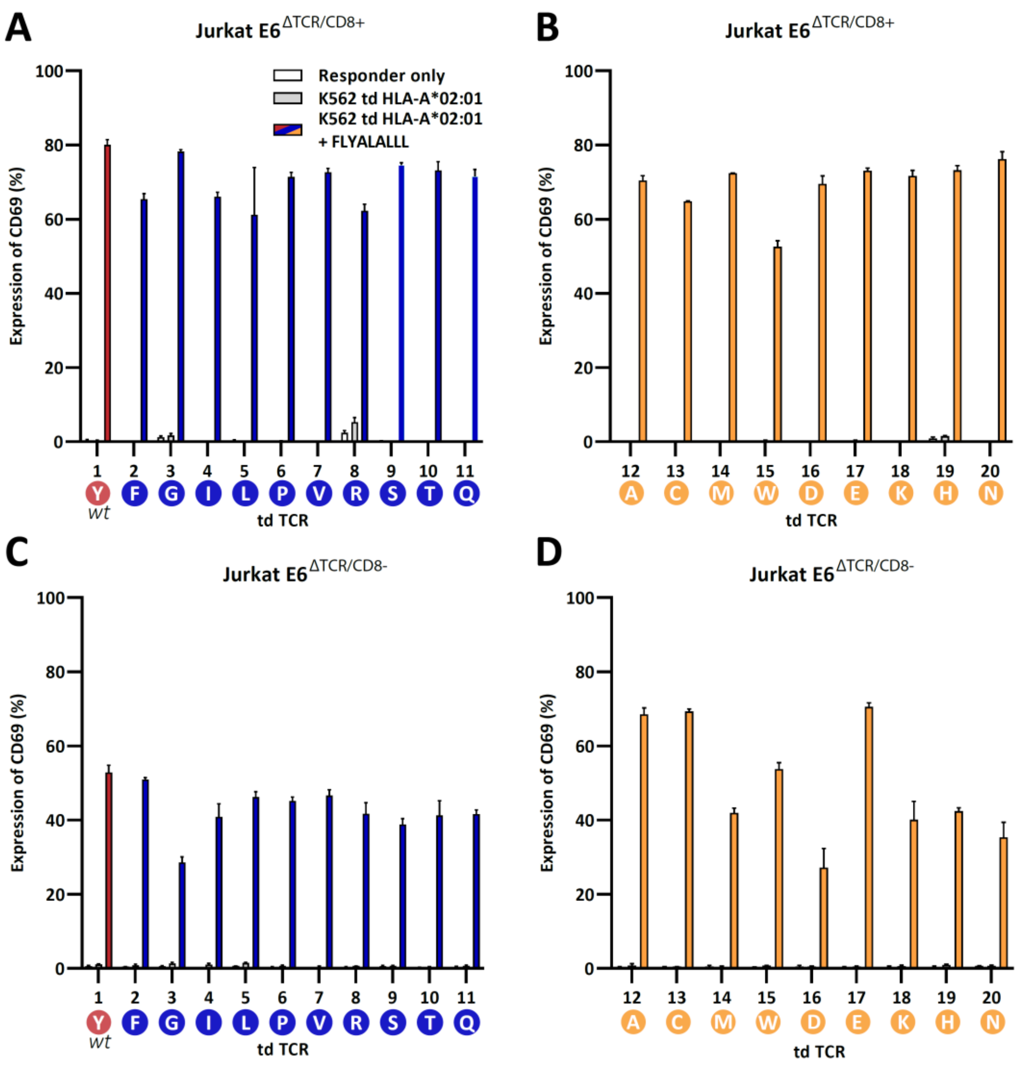

Supplementary Figure 6. TCR gene transfer introduced EBV-LMP2^{FLY} specificity and reactivity into Jurkat E6 cells. The endogenous TCR of Jurkat E6 cells was knocked out using Crispr-Cas9 technology (ΔTCR). Jurkat E6$^{\Delta TCR}$ cells were transduced with CD8 to simulate CD8^{pos} T cells (Jurkat E6$^{\Delta TCR/CD8+}$). Transduced cells were purified based on expression of murine-TCR-Cβ using MACS and expanded. HLA-A*02:01-transduced K562 cell-lines were pulsed with 10^{-6} M of EBV-LMP2^{FLY}-specific peptide. **A** and **B**) EBV-LMP2^{FLY}-specific TCR-transduced Jurkat E6$^{\Delta TCR/CD8+}$ cells were tested for recognition of K562 cells transduced with HLA-A*02:01 and pulsed with and without EBV-LMP2^{FLY} peptide. The wildtype EBV-LMP2^{FLY}-specific TCRαβ-sequence is shown in red, additional highly-similar TCRβ-sequences found *ex vivo* are shown in blue (**A**). TCRβ-sequences not found *ex vivo* are shown in orange (**B**). **C** and **D**) CD8 co-receptor dependency was assessed by testing EBV-LMP2^{FLY}-specific TCR-transduced Jurkat E6$^{\Delta TCR/CD8-}$ for recognition of K562 cells transduced with HLA-A*02:01 and pulsed with and without EBV-LMP2^{FLY} peptide. The wildtype EBV-LMP2^{FLY}-specific TCRαβ-sequence is shown in red, additional highly-similar TCRβ-sequences found *ex vivo* are shown in blue (**C**). TCRβ-sequences not found *ex vivo* are shown in orange (**D**).

Shown are means with standard deviations of one experiment carried out in triplicate
Abbreviations: td; transduced, wt; wildtype
